# Supplementary material for: What is a supercoiling-sensitive gene? Insights from topoisomerase I inhibition in the Gram-negative bacterium Dickeya dadantii
Source: Nucleic Acids Res. 2022 Aug 11;50(16):9149–61. doi: 10.1093/nar/gkac679 (PMC9458453; doi:10.1093/nar/gkac679)
Supplement: gkac679_Supplemental_Files [file gkac679_supplemental_files.zip › Supplementary_material_V2.pdf]

What is a supercoiling-sensitive gene?
Insights from topoisomerase I inhibition
in the Gram-negative bacterium *Dickeya dadantii*

Supplementary Information

Maïwenn Pineau, Shiny Martis B., Raphaël Forquet, Jessica Baude, Camille Villard, Lucie Grand, Florence Popowycz, Laurent Soulère, Florence Hommais, William Nasser, Sylvie Reverchon and Sam Meyer

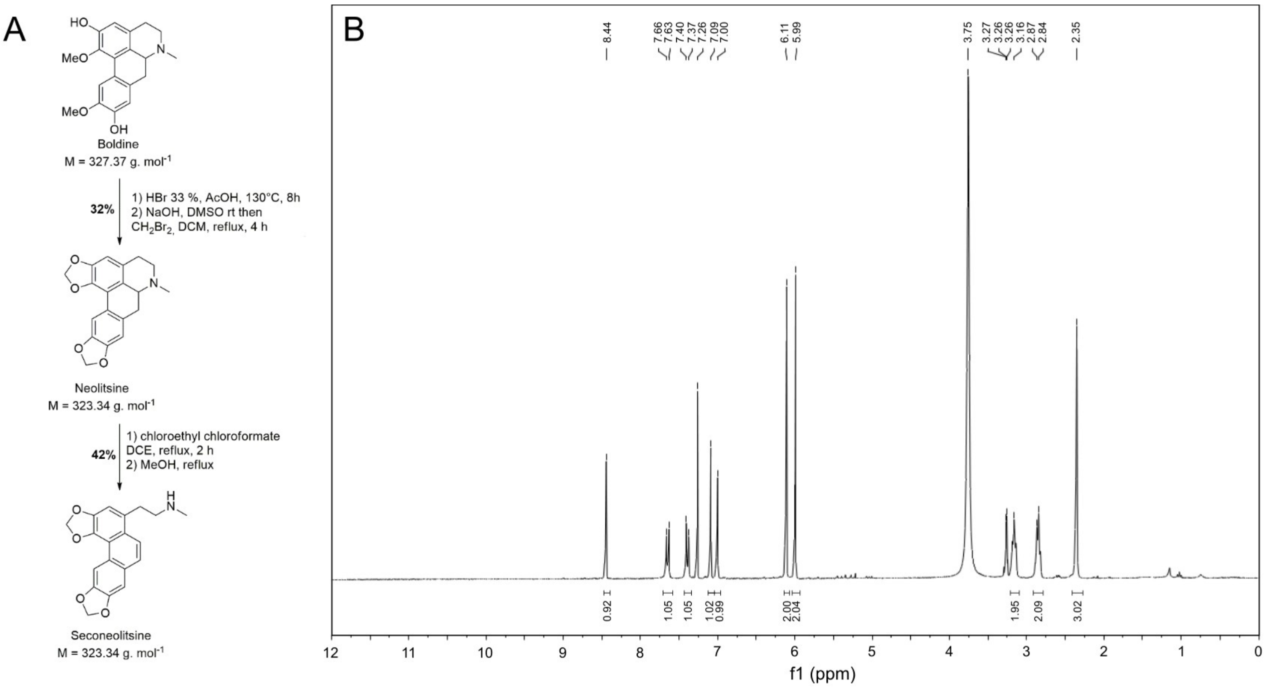

Fig. S1: (A) Synthesis of seconeolitsine from boldine. (B) Purity of the product measurement: spectrum of the compound by proton nuclear magnetic resonance (^{1}H NMR) (300MHz in $CDCl_3$ / CD_3OD).

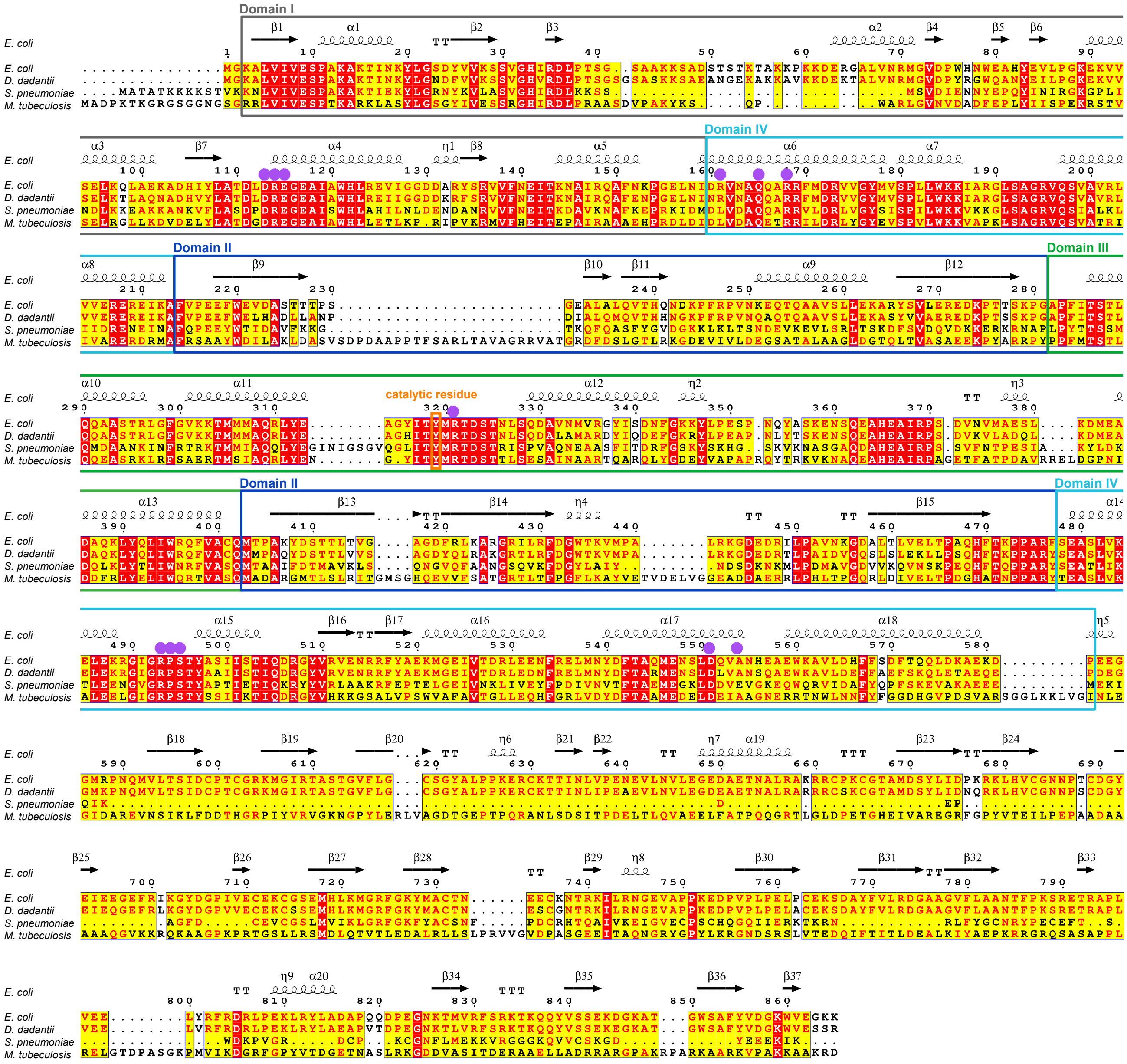

Fig. S2: Alignment of *E. coli*, *D. dadantii*, *S. pneumoniae* and *M. tuberculosis* topoisomerase I sequences obtained using ENDscript (1). Identities are highlighted in red, similarities in yellow. Secondary structure elements of *E. coli* topoisomerase I are shown above the sequences (α-helices by squiggles, β-strands by arrows, strict α-turns by TTT letters and β-turns by TT letters). The catalytic tyrosine residue is shown by an orange box (Tyr-319 for *E. coli*). Residues indicated with purple dots are part of the nucleotide-binding site and might interact with seconeolitsine. Grey, dark blue, green and light blue boxes indicate previously described topoisomerase I domains (2).

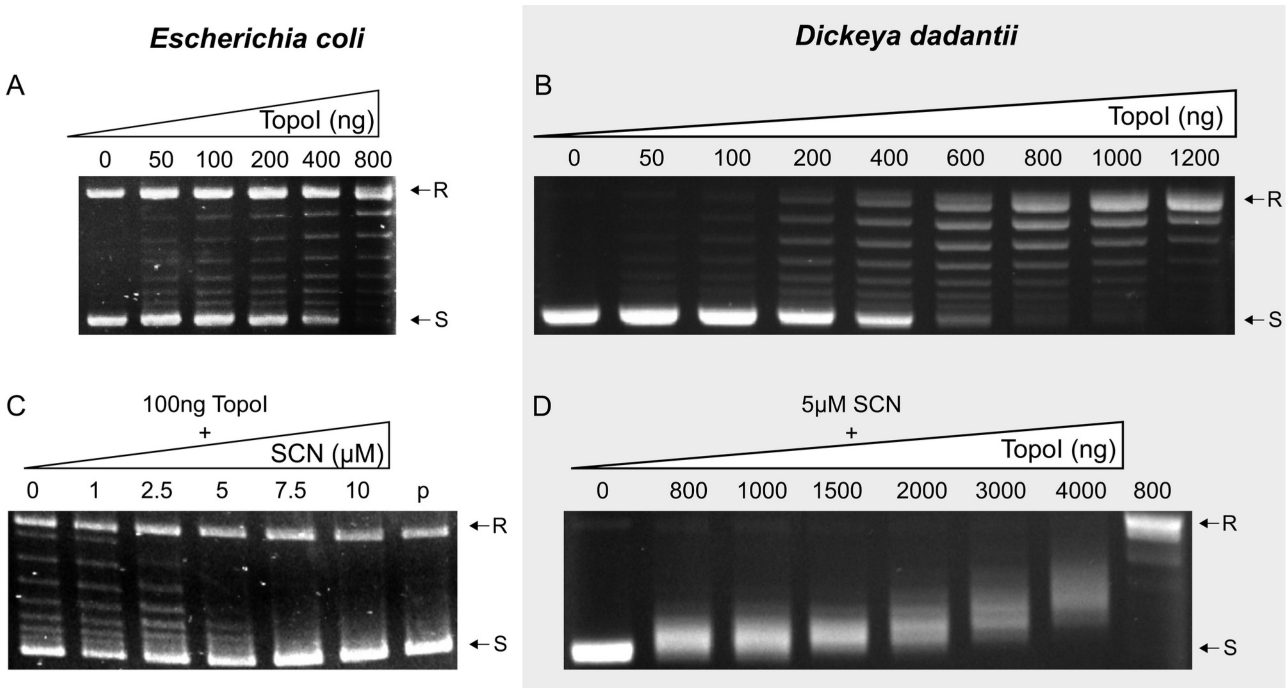

Fig. S3: Activity of purified topoisomerase I from *E. coli* (A) and *D. dadantii* (B). 0.5 µg of pUC18 plasmid was incubated with the indicated mass of topoisomerase I for 15 min at 37°C. R corresponds to pUC18 relaxed form, S to the highly negatively supercoiled form. Inhibition of *E. coli* (C) and *D. dadantii* (D) topoisomerase I by seconeolitsine. The indicated amount of seconeolitsine was first incubated 10 min with 100 ng *E. coli* topoisomerase I (C) or the indicated amount of *D. dadantii* topoisomerase I (D) at 4°C. 0.5 µg of pUC18 plasmid (p) was then added and the mix was incubated for 15 min at 37°C. In (D), the lane at the right of the gel contained no seconeolitsine and 800 ng topoI. The IC50 of seconeolitsine for *E.coli* topoisomerase I was estimated around 7 µM (see Materials and Methods).

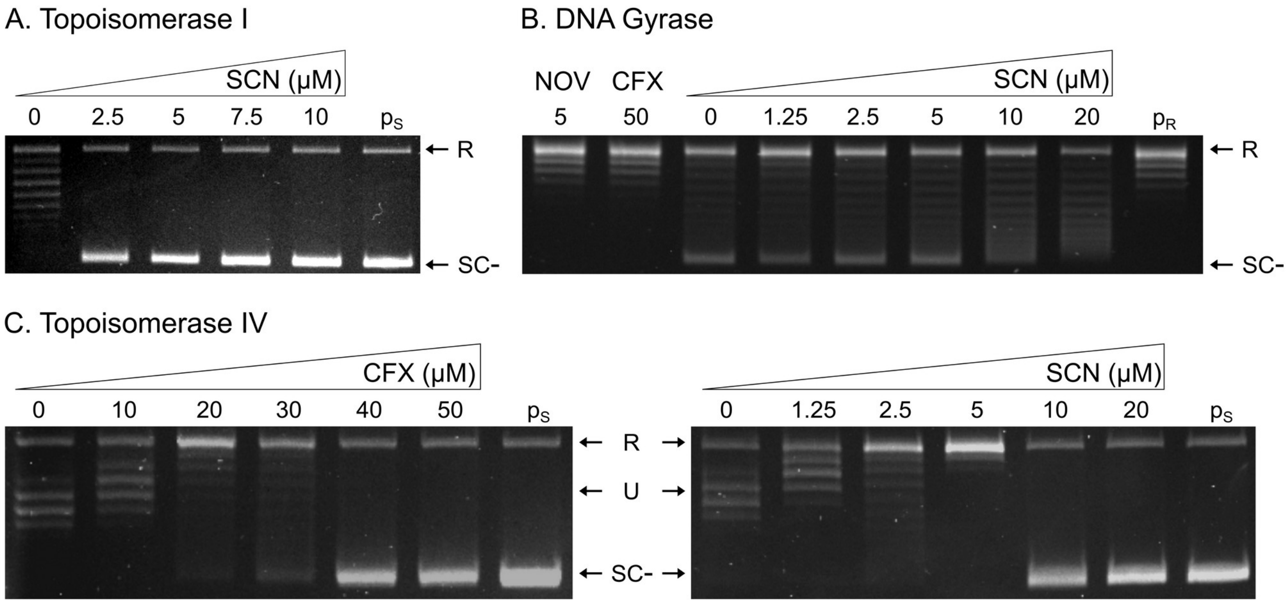

Fig. S4: Activity of purified *D. dadantii* topoisomerase I (A), *E. coli* DNA gyrase (B) and *E. coli* topoisomerase IV (C) inhibited by novobiocin (NOV), ciprofloxacin (CFX, a gyrase and topoIV inhibitor used as control) or seconeolitsine (SCN). Agarose gels of supercoiled (pS) or relaxed (pR)

pBR322 plasmids were treated following manufacturer's instructions (Inspiralis kits), with the indicated amount of inhibitor. S indicates the negatively supercoiled form, R the relaxed form, and U the (unknown) form obtained after topoIV treatment without inhibitor. With the employed pBR322 plasmids, topoI is fully inhibited by less than 2.5 µM seconeolitsine. DNA gyrase is almost unaffected (slight reduction of the most supercoiled topoisomers at the highest concentrations of seconeolitsine). TopoIV is inhibited by seconeolitsine with an IC50 between 5 and 10 µM; note that the U form migrates as weakly positively supercoiled plasmids, so that a partial topoIV inhibition (by low concentrations of CFX or SCN) results in more relaxed plasmids (remaining at the top of the gel), whereas a full inhibition by either drug results in highly negatively supercoiled plasmids (bottom of the gel, like the untreated plasmids). The IC50 of topoIV is around three-fold higher than the IC50 of topoI.

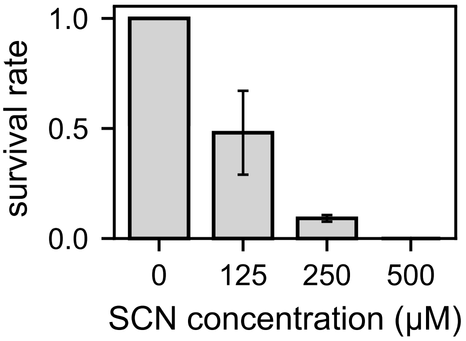

Fig. S5: Inhibition of the growth of *E. coli* NM522 cells by seconeolitsine. Each bar indicates the proportion of growing colonies on plates with the specified amount of seconeolitsine compared to plates without seconeolitsine, with a 95% statistical confidence interval.

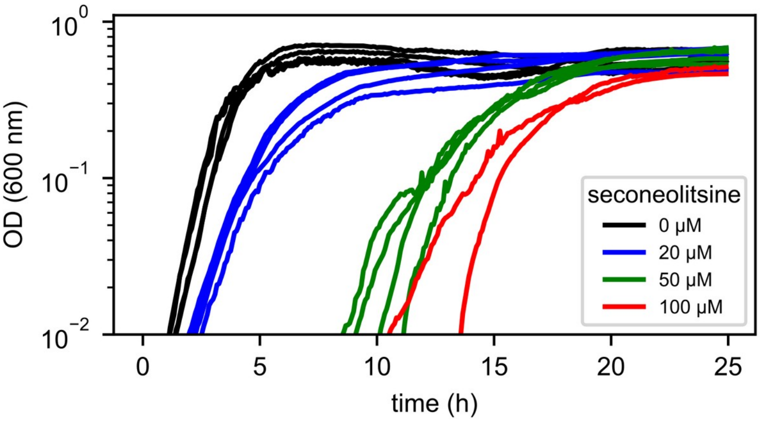

Fig. S6: Growth curves of the Gram-positive bacterium *B. subtilis* in presence of increasing amounts of seconeolitsine, solvated in a constant (5% vol) volume of DMSO.

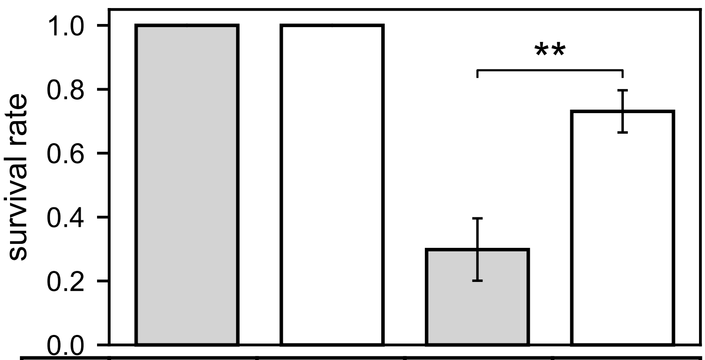

SCN	-	-	+	+
IPTG	-	+	-	+
topoI	+	++	+	++

Fig. S7: Effect of topoisomerase I expression level on survival rate in presence of seconeolitsine. The *D. dadantii* topoisomerase I gene under the control of an IPTG-inducible promoter was inserted into pQE80L and transformed into *E. coli* NM522 cells. The survival rate is defined as the proportion of growing colonies observed on plates containing 125 µM seconeolitsine vs without seconeolitsine. In the absence of IPTG, *D. dadantii* topoisomerase I is only expressed at a basal level and the survival rate on plates containing seconeolitsine is around 30%. In the presence of 0.5 mM IPTG, *D. dadantii* topoisomerase I is overexpressed and the survival rate is significantly higher (73%, *P*=0.0015).

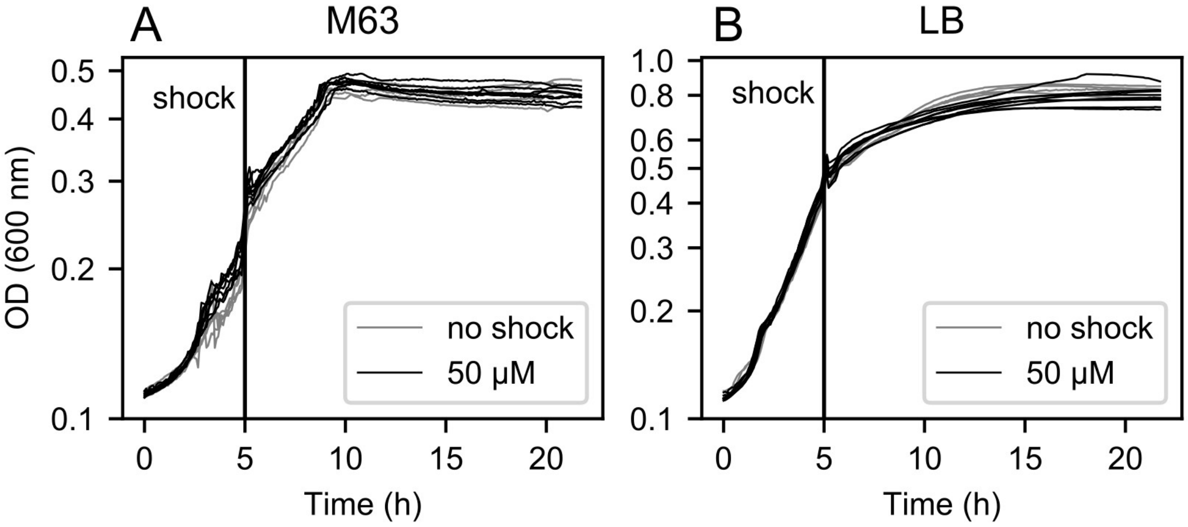

Fig. S8: Growth curves of *D. dadantii* treated with a seconeolitsine shock at 50 µM during exponential growth (vertical line), exhibiting no significant effect (multiple black and grey lines indicate replicates of treated and untreated samples, respectively, and are almost indistinguishable). The chosen concentration thus minimises the metabolic effect of the shock, while inducing a significant increase in SC (Fig. 3). The experiment was carried in M63+glucose (A) and LB (B) culture media.

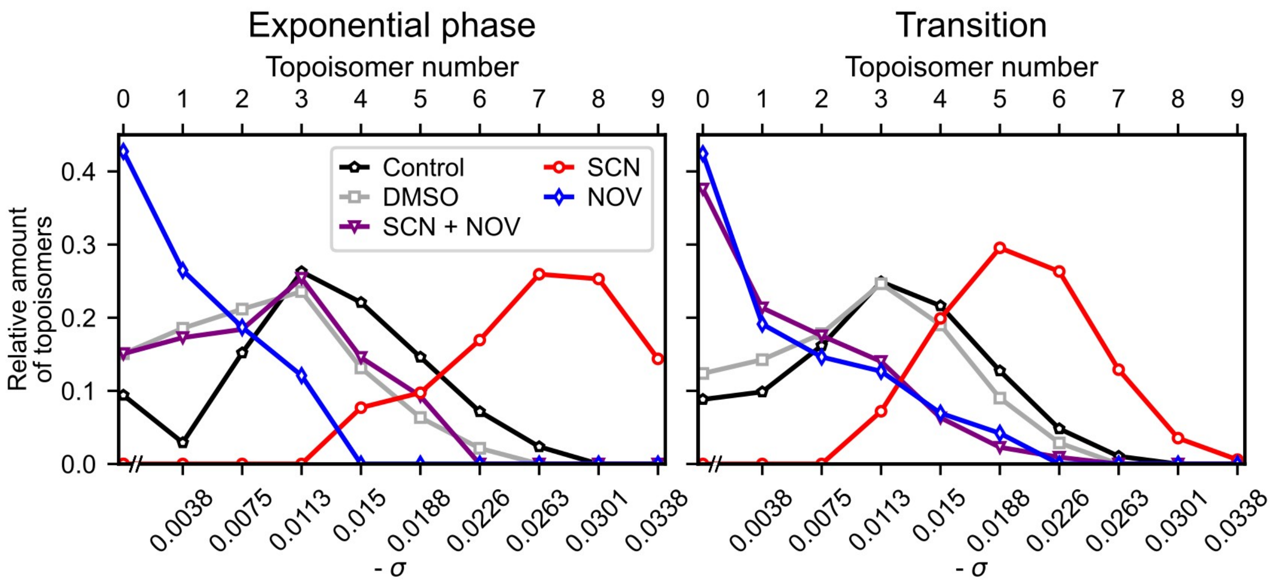

Fig. S9: Quantification of topoisomer distribution of pUC18 plasmids isolated from *D. dadantii.* Cells were treated with seconeolitsine (50 µM), novobiocin (100 µg.ml^{-1}) or both, in exponential (left) and transition to stationary phase (right). The most relaxed fractions were not fully resolved on the chloroquine gel, hence the discontinuity indicated in the x-axis.

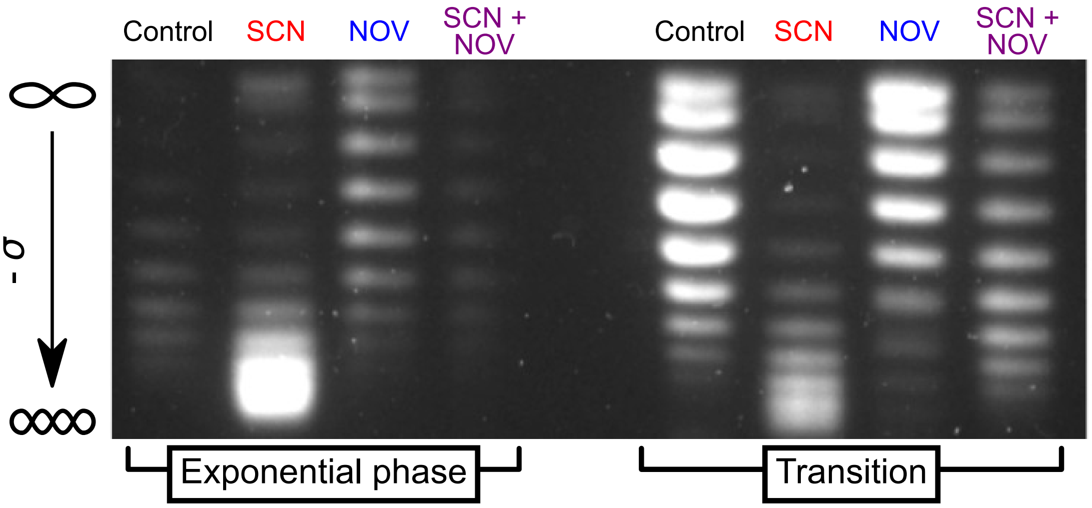

Fig. S10: Agarose-chloroquine gels of pUC18 plasmids isolated from *E. coli* NM522 cells in the exponential phase (left) or at the transition to stationary phase (right), 15 min after antibiotic shocks (seconeolitsine 50 µM, novobiocin 100 µg.ml^{-1}, combination of both, from left to right). The downward migration increases with the negative SC level. The results are qualitatively similar to those obtained in *D. dadantii*, with a stronger effect of seconeolitsine.

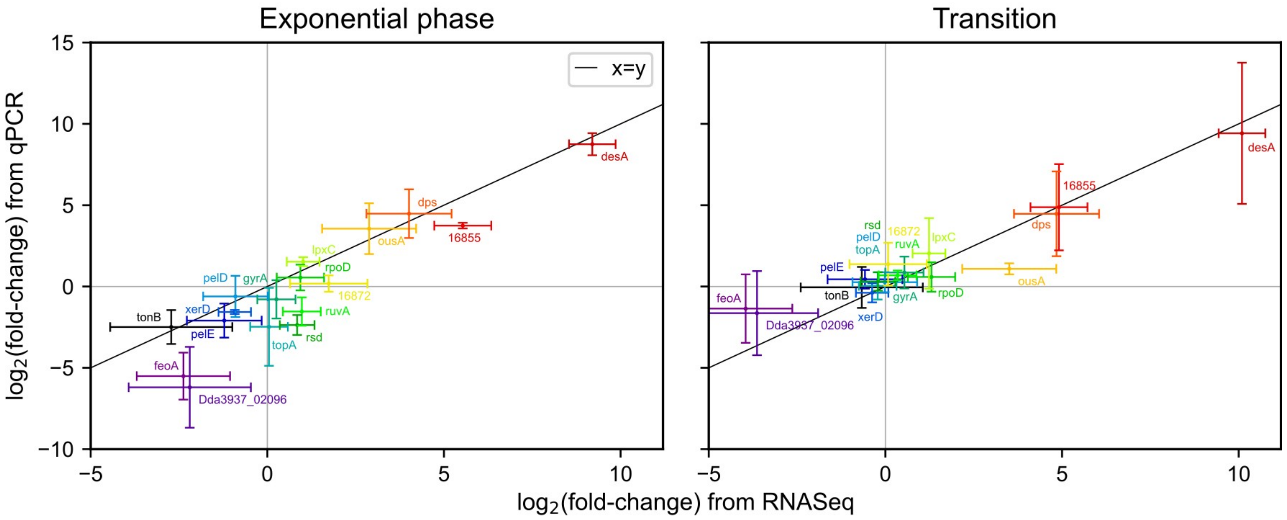

Fig. S11: Comparison of gene responses obtained with qRT-PCR and RNA-Seq to seconeolitsine treatment in exponential (left) and transition to stationary phase (right). Pearson's correlation coefficients reached 0.904 with p-value of $6.23e^{-7}$ (exponential phase) and 0.917 with p-value of $2.32e^{-7}$ (transition).

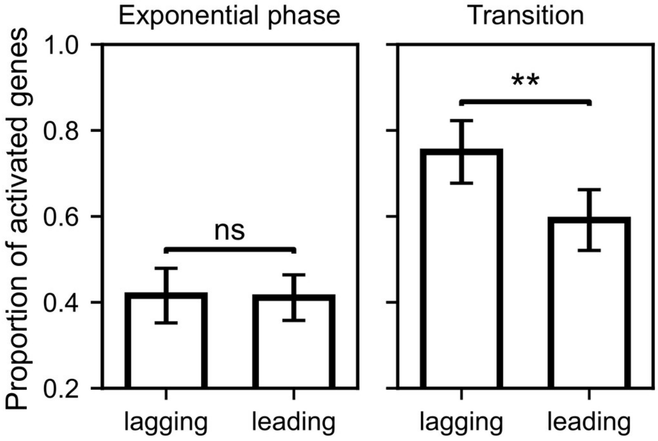

Fig. S12: Proportion of activated genes (among differentially expressed ones) on the lagging vs leading replicative strand in exponential phase and at the transition to stationary phase (*P*=0.0015).

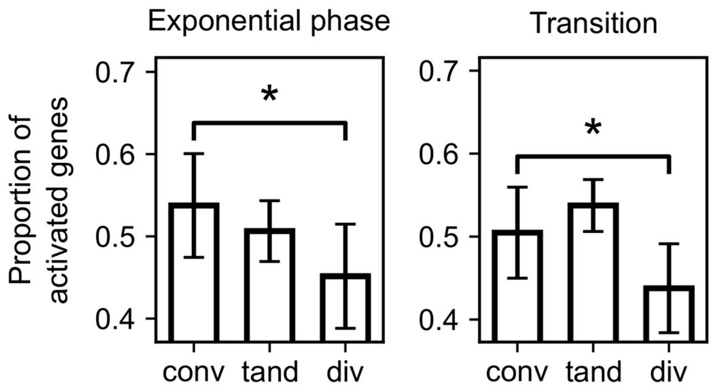

Fig. S13: Proportion of activated genes (among differentially expressed ones) on convergent, tandem and divergent genes following novobiocin treatment in *D. dadantii* in exponential phase (P=0.030) and at the transition to stationary phase (P=0.044) (3).

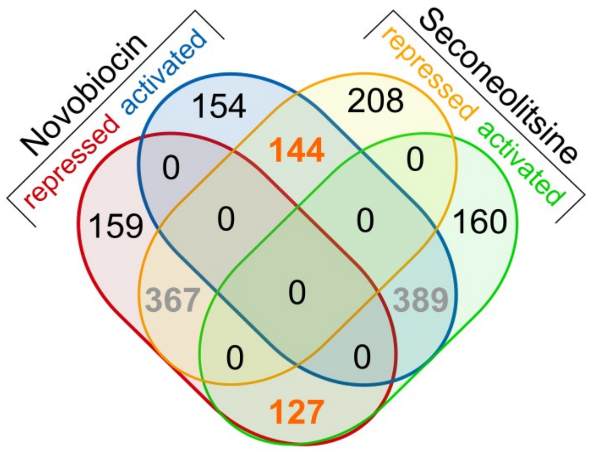

Fig. S14: Venn diagram of the response of *S. pneumoniae* genes to novobiocin and seconeolitsine treatments in exponential phase, computed from the data of (4). The number of genes responding in opposite directions to the two drugs are indicated in orange, and those in the same direction in gray.

Tab. S1: List of primers used in PCR.

Gene / plasmid name	Accession number	Forward primer	Reverse primer
ryhB	Dda3937_04678	GACTGAGAATTCGCGTTCCGGGAAAGC CAAA	CATAGCATGCTAAAAAAAAGCCAGCAC CCGAGC
tonB	Dda3937_00565	GACGCCAGAACCGGAAATAC	CGTTTCCTTCGGTGGTTCC
ousA	Dda3937_00791	GCAACACGTGGAGAAACTGG	CTTGGCATGTAGGTCAGCAG
ruvA	Dda3937_01621	CGGCGTCGGATATGAAGTTC	CACGCCGTTCACTTTGATCA
rsd	Dda3937_00236	ACGCTGGATGAATTTTGCCA	ATTTGCTGGGTGTTGCTACG
dps	Dda3937_02884	ACACGAGATGCTGGATACCT	CGTTGTGGATATCAAGCGGG
xerD	Dda3937_02296	GCTGCCGAAAGATCTGACG	TGTTGCCCTTCCCTATCACG
desA	Dda3937_00786	CCATCAGCCAGCAAACCTAC	GCGAAGGTCAGATCCAGTTG
	Dda3937_01473	GGCGGAACAGAACCTTAACC	CGCTTTCACGTACGGATACC
	Dda3937_01484	AACGGAACAAGATCAGCTGC	AGGTCATACAGCTCGTTCCA
lpxC	Dda3937_02232	ACGGTAAGCGCATTCTGGTA	CATCCAGCAGCAGGTAGACA
pelD	Dda3937_03372	TTGTGGAAGGTAACGCGCAGTTTG	ATGGCAAATTCACCAACGGCTCTC
pelE	Dda3937_03371	AGCGAATTCAAAGCAGCACT	GGCGTTTCGATGTACAGGTT
topA	Dda3937_00587	GAGCAAGAAAAGCGCTGAAG	TTGCGAGATAGACGTGATCG
rpoD	Dda3937_03534	CCGACCGCTATTGATGAAAT	CACAGTTCGACGCACAGTTT
gyrA	Dda3937_01774	GGTAAATATCACCCGCATGG	TCTCCAGATCGGAAAGCAGT
feoA	Dda3937_02095	GGGGTT TCAGAAAGGATCGG	AGAATCAAAATCTGCGCTTCG
	Dda3937_02096	TCAAACGCTGCT TGATCTCC	TTGCCCATCTCCCAGTTG AT
rpoA	Dda3937_01515	AAACCGCGCCTGGTAGATA	CCTTTCAGGTTGAGCAGGAT
topA	Dda3937_00587	CACCATCACCATCACGGATCCATGGGTA AAGCTCTCGTTATCGTC	AAGCTCAGCTAATTAAGCTTCTCGAGTT AGCGGCTGCTTTCCACCCAC
pQE80L		AAGCTTAATTAGCTGAGCTTGGACTCC	GGATCCGTGATGGTGATGGTGATGCGAT CC

Tab. S2: Lists of *D. dadantii* genes responding to seconeolitsine treatment in exponential and transition to stationary phase.

Tab. S3: Lists of *D. dadantii* genes (locus tags and gene names or ASAP identifiers (5)) significantly affected by seconeolitsine and novobiocin treatment.

References

1. Robert,X. and Gouet,P. (2014) Deciphering key features in protein structures with the new ENDscript server. *Nucleic Acids Res.*, 42, W320-324.

2. García,M.T., Blázquez,M.A., Ferrándiz,M.J., Sanz,M.J., Silva-Martín,N., Hermoso,J.A. and de la Campa,A.G. (2011) New alkaloid antibiotics that target the DNA topoisomerase I of Streptococcus pneumoniae. *J. Biol. Chem.*, 286, 6402–6413.

3. El Houdaigui,B., Forquet,R., Hindré,T., Schneider,D., Nasser,W., Reverchon,S. and Meyer,S. (2019) Bacterial genome architecture shapes global transcriptional regulation by DNA supercoiling. *Nucleic Acids Res.*, 47, 5648–5657.

4. Ferrándiz,M.-J., Martín-Galiano,A.J., Arnanz,C., Camacho-Soguero,I., Tirado-Vélez,J.-M. and de la Campa,A.G. (2016) An increase in negative supercoiling in bacteria reveals topology-reacting gene clusters and a homeostatic response mediated by the DNA topoisomerase I gene. *Nucleic Acids Res.*, 44, 7292–7303.

5. Glasner,J.D., Liss,P., Plunkett,G., Darling,A., Prasad,T., Rusch,M., Byrnes,A., Gilson,M., Biehl,B., Blattner,F.R., *et al.* (2003) ASAP, a systematic annotation package for community analysis of genomes. *Nucleic Acids Res.*, 31, 147–151.
